# Transnational multistakeholder partnerships for sustainable development: Conditions for success

**DOI:** 10.1007/s13280-015-0684-2

**Published:** 2015-07-23

**Authors:** Philipp Pattberg, Oscar Widerberg

**Affiliations:** Department of Environmental Policy Analysis, Institute for Environmental Studies, VU University Amsterdam, De Boelelaan 1087, 1081 HV Amsterdam, The Netherlands

**Keywords:** Multistakeholder partnerships, Sustainable development, Climate change, Global governance, Sustainable development goals

## Abstract

This perspective discusses nine conditions for enhancing the performance of multistakeholder partnerships for sustainable development. Such partnerships have become mainstream implementation mechanisms for attaining international sustainable development goals and are also frequently used in other adjacent policy domains such as climate change, health and biodiversity. While multistakeholder arrangements are widely perceived as a positive contribution to addressing global change, few studies have systematically evaluated the existing evidence for their positive performance. This poses an urgent and important challenge for researchers and practitioners to understand and improve the effectiveness of partnerships, in particular since their popularity increases despite their past track record. The recommendations presented are based on own research, a literature survey and discussions with a large number or international Civil Society Organizations at two occasions during 2014. This article proceeds as follows: first, we define multistakeholder partnerships, outline their rational and summarize available assessments on partnership success; second, we provide a set of concrete recommendations based on lessons-learned from over 10 years of scholarship; and third, we conclude with some reflections on the future of multistakeholder governance for sustainability.

## Introduction

As decision-makers continue to struggle with providing adequate solutions to pressing global environmental challenges such as climate change, biodiversity loss, deforestation, and natural disasters, calls for innovative approaches to ‘navigate the Anthropocene’ (Biermann et al. 2012), which challenge the hierarchical state-led model of governance (Hajer et al. 2015) are getting louder. Proponents argue that coalitions and cooperation between government agencies, business actors, and civil society will increase the likelihood to stay within a ‘safe operating space for humanity’ (Rockström et al. 2009) as new collaborative arrangements are expected to forge more efficient, effective, and inclusive responses to global policy problems. In the area of climate change, for example, recent scholarship has scrutinized the emergence of a loosely coupled regime complex (Biermann et al. 2009; Keohane and Victor 2011; Zelli 2011) that shows features of a polycentric governance architecture (Cole 2015). A particularly popular arrangement within this broader trend has been multistakeholder partnerships, which have played a crucial role in implementing sustainable development goals ever since the 2002 World Summit on Sustainable Development in Johannesburg. More than 340 partnerships for sustainable development were consequently registered at the United Nations (Andonova and Levy 2003), and the ‘partnership approach’ is currently being emulated in many other issue areas of global governance, such health, water governance, and climate change.

While bottom-up transnational multistakeholder arrangements are widely perceived as a potential contribution to addressing global change, recent studies find little evidence for positive performance. This poses an urgent and important challenge for researchers and practitioners to understand and improve the effectiveness of partnerships, in particular, since their popularity only seems to increase despite their mixed track record. It is also a particularly timely quest given that the year 2015 comprises high-profile negotiations taking place on the Post-2015 Development Agenda: the Hyogo Framework of Action on natural hazards and the United Nations Framework Convention on Climate Change (UNFCCC). Within the context of the latter, demand for a more structured engagement of the UNFCCC with bottom-up transnational arrangements is building up (Chan and Pauw 2014; Widerberg and Pattberg 2015).

In this perspective, we argue that lessons learned from evidence-based assessments of transnational multistakeholder partnerships should urgently be taken into account when designing or re-designing existing transnational multistakeholder arrangements. We identify nine conditions for improved performance arranged across three overarching themes: actors (leadership, partners); processes (goal setting, funding, management, monitoring); and contexts (meta-governance, problem structure, and sociopolitical contexts). The nine conditions have been identified by carrying out a systematic review of research on transnational multistakeholder partnerships in the field of sustainable development. The nine conditions have been distilled from the literature by clustering the explanatory factors for success or failure in transnational multistakeholder partnerships identified from the scholarly literature. The review has been complemented by input from some of the world’s largest Civil Society Organizations (CSOs), to which we presented our study and discussed the results at two separate occasions during 2014: first at a workshop with CSO Strategy Directors collaborating under the umbrella of the International Civil Society Center (ICSC); and second, during the 2014 Global Perspectives conference arranged by the ICSC in collaboration with the Organisation for Economic Co-operation and Development (OECD).

This article is structured as follows. The first section defines transnational multistakeholder partnerships, provides a brief history of their emergence, and assesses their performance to date. In the second section, we identify nine conditions for success of multistakeholder arrangements and provide policy-advice. The final section concludes with some reflections on the future role and relevance of polycentric bottom-up governance for sustainability.

## Transnational multistakeholder partnerships: Definition, rationale, and past performance

### Definition

Identifying the precise unit of analysis when assessing the performance of transnational partnerships is challenging. Practitioners and scholars have used the term ‘partnership’ to describe just about any type of collaboration between state and non-state actors. Also the vast and growing literature on public–private partnerships suffers from conceptual confusion, competing definitions, disparate research traditions, and a normative and value-laden agenda of promoting partnerships. This state of conceptual vagueness has led some scholars to describe the term partnership as “conceptually empty and merely politically expedient” (Brinkerhoff and Brinkerhoff 2011, p. 31). Despite the lack of a broadly agreed definition, most scholars agree about the constitutive features of multistakeholder partnerships at the global level, most notably: transnationality (involving cross-border interactions and non-state relations); public policy objectives (as opposed to public “bads” or exclusively private “goods”); and a network structure (coordination by participating actors rather than coordination by a central hierarchy). Following Schäferhoff et al. (2009, p. 455), we define transnational multistakeholder partnerships as “institutionalized transboundary interactions between public and private actors, which aim at the provision of collective goods.” Finally, we understand ‘performance’, ‘success,’ and ‘effectiveness’ in terms of problem-solving capacity of partnerships to address the issue they have set out to solve.

### The rationale behind multistakeholder partnerships

Multistakeholder partnerships at the global level emerged during the 1990 s as a new and innovative governance tool vis-à-vis traditional intergovernmental cooperation through treaty-making and have become part and parcel of many countries’ developmental strategies. They are now being employed as governance instruments in issue areas ranging from environment, health, and development cooperation to social rights and security (Schäferhoff et al. 2009). The emergence of transnational multistakeholder partnerships can be traced back to the 1992 Earth Summit, where Agenda 21 called for a “Global Partnership for Sustainable Development” and alluded to multistakeholder partnerships between “public, private and community sectors” to boost implementation (UNCED 1992). A decade later, the World Summit on Sustainable Development (WSSD) in Johannesburg reiterated the message, and the so called Type II or Johannesburg partnerships were created. More recently, in 2012, at the United Nations Conference on Sustainable Development (Rio + 20), the central role of partnerships was emphasized in the outcome document: “The Future We Want” (Pattberg and Mert 2013). Consequently, multistakeholder partnerships have become integral to global environmental governance from the perspective of governments and are likely to remain so in the implementation of the SDGs, the emerging new climate change regime and other issues areas.

Advocates of multistakeholder partnerships emphasize their flexible, adaptive, and decentralized nature, whereas critics object to the market-based narrative and argue that partnerships are a neoliberal construction invented to increase the power of private interest in global affairs, in particular in the developing world (Zammit 2003). Some even claim that UN-sponsored partnerships are a way to invite special interest into the UN, boost corporatism, and allow the private sector to make use of the UN’s good name while merely paying lip-service to the goals they set out. For example, the UN’s Global Compact, which is the UN’s high-profile corporate governance partnership, has repeatedly been accused of “blue-washing” meaning that corporations make use of the UN’s good name by signing up to a number of principles they never intend to follow (Bruno and Karliner 2000). What is more, some developing countries have also been weary of giving partnerships too much attention and consequently accused developed nations of shifting responsibility for funding away from traditional Official Development Assistance (ODA).

While debates about the pros and cons of multistakeholder partnerships continue, they have proliferated in a number of issue areas. The empirical evidence of this trend is the found in multiple existing registries and databases on partnerships. A well-studied registry was set up in conjunction with the 2002 WSSD and administered by the UN’s Commission on Sustainable Development (CSD). At its peak, it included over 340 entries (today that number is down to 196^1^). The first registry has been succeeded by the SD in Action Registry which applies a somewhat broader definition of partnerships than the original registry, currently listing 1400 voluntary actions and commitments. A more recent registry was set up in 2014 after the Third International Conference for Small-Island Developing States (SIDS) where nearly 300 multistakeholder partnerships were announced. Interestingly, a similar process is taking place in the climate-change regime where a web-portal for Cooperative Initiatives has been created to show case climate initiatives submitted by countries and observers to the Secretariat to the United Nations Framework Convention on Climate Change (UNFCCC) (UNFCCC 2014). Mirroring the SD in Action Registry, a homepage for Non-state Actor Zone for Climate Action (NAZCA) was launched in end 2014 at the 20th Conference of the Parties (COP) to the UNFCCC with over 1000 entries of “Cooperative and Individual Actions on Climate Change in Partnership with Countries” (UNFCCC 2015). A large number of the actions registered in the sustainable development and the climate change registries do not qualify as partnerships according to our definition; however, it serves as a clear indication of new governance mechanisms increasingly being applied by governments.

### Key findings on the past performance of multistakeholder partnerships

In the context of an increased use of transnational multistakeholder partnerships in global environmental governance, a key concern for policy-makers and academics alike is their overall limited effectiveness (Andonova and Levy 2003; Zammit 2003; Hale and Mauzerall 2004; Börzel and Risse 2005; Glasbergen et al. 2007; Bitzer et al. 2008; Vollmer 2009; Andonova 2010; Pattberg 2010; Bäckstrand 2012; Pattberg et al. 2012; Beisheim and Liese 2014). Individual partnerships such as the GAVI Alliance that enhances the dissemination of immunization or the standard-setting Forest Stewardship Council (FSC) have proven highly effective in problem-solving (Pattberg 2005; Pattberg 2007; Beisheim and Liese 2014). On the whole, however, concluding from recent analyses of the WSSD sample, partnerships have a limited track-record in terms of effectiveness (Schäferhoff et al. 2009; Pattberg et al. 2012). The analysis of WSSD Partnerships draws four conclusions.

**Fig. 1:**
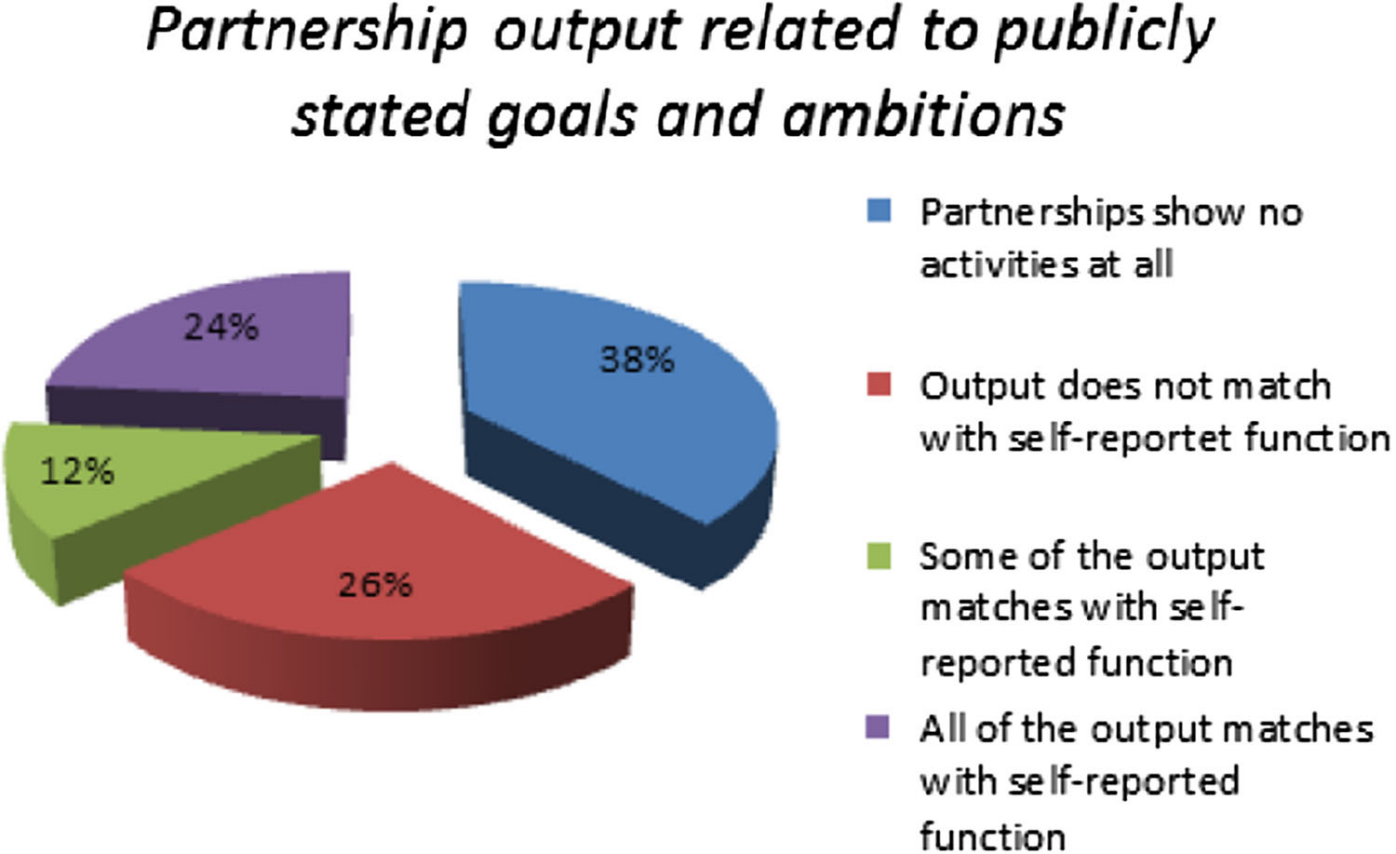
Partnership output related to publicly stated goals and ambitions (*Source* Pattberg et al. 2012 and own calculations)

First, on analyzing the sample of 340 partnerships after more than five years since inception, approximately 38 percent show low levels or no measurable output. Moreover, roughly 42 percent (86) of the partnerships with measurable output engage in activities without direct relation to their publicly stated goals and ambitions (see Fig. 1). Summing up, of these numbers, 211 partnerships are inactive, lack any outputs, or fail to match their stated ambition with their observed activities (see Fig. 1).

**Fig. 2:**
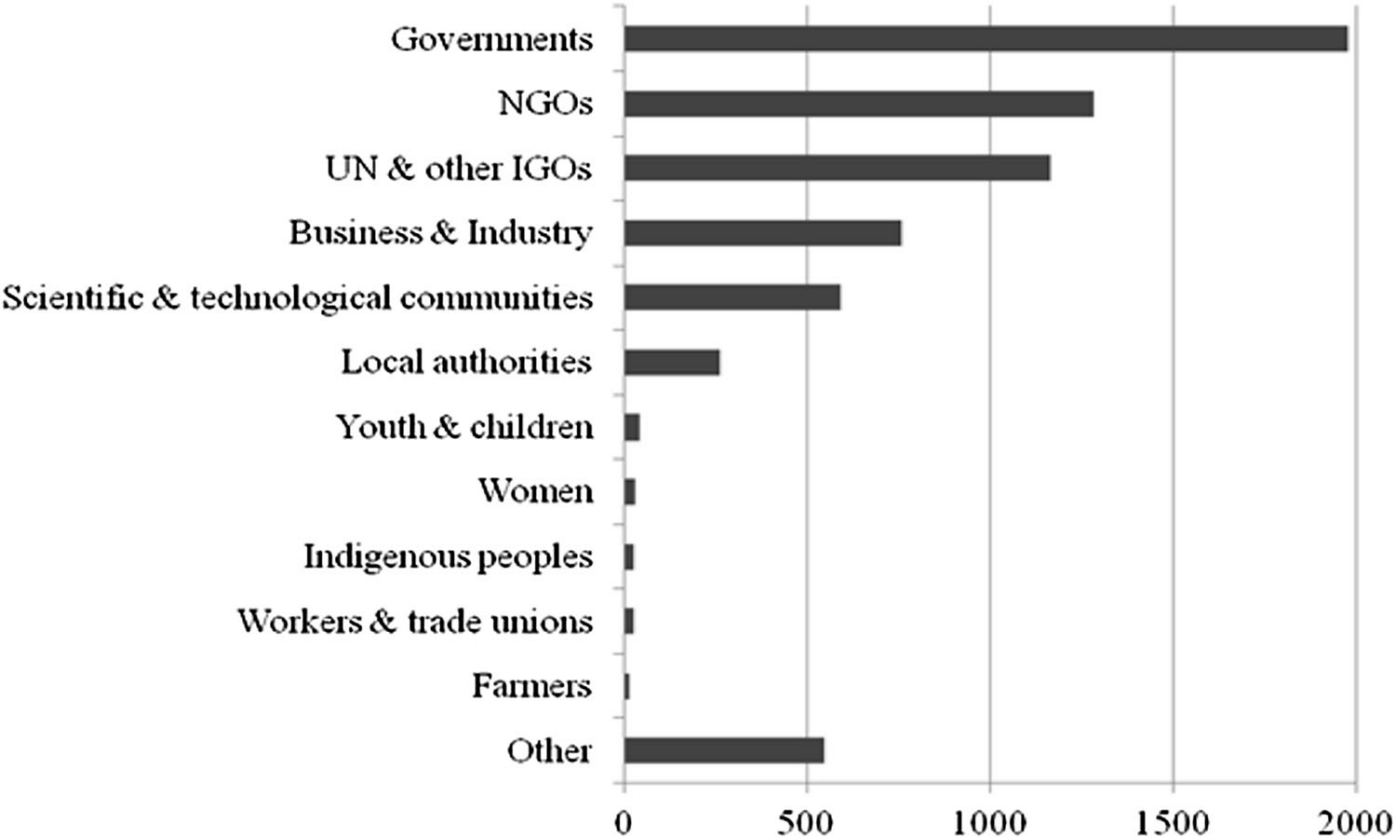
Number of partners from different sectors (*Source* Pattberg et al. 2012)

**Fig. 3:**
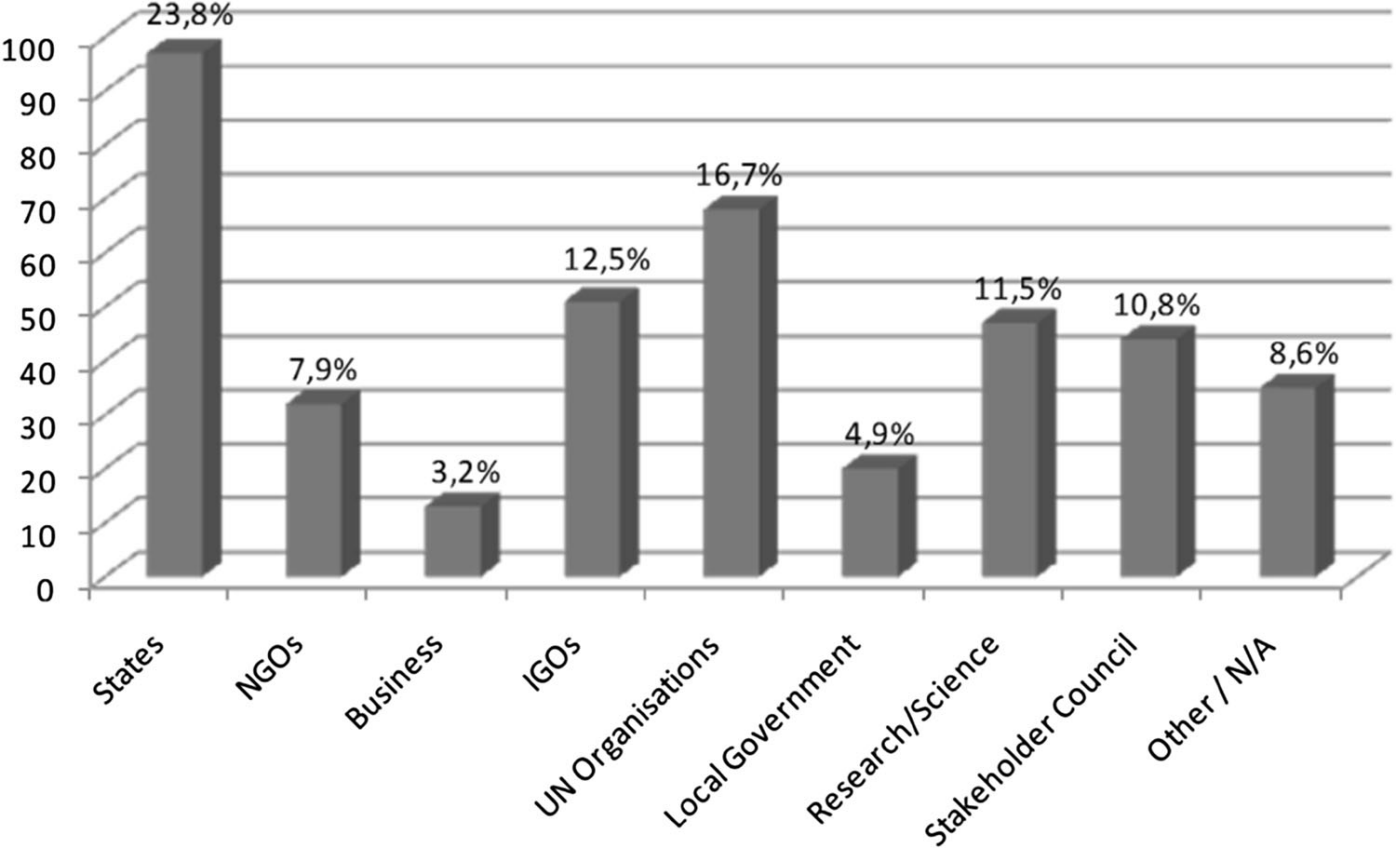
Number of partnerships led by partners from a specific sector (*Source* Pattberg et al. 2012)

Second, partnerships fail to deliver on the promises rehearsed by many of their advocates. They are not filling governance gaps left open by governments with new norms. Nor do they foster implementation of existing intergovernmental regulations to a significant degree. Finally, partnerships fail to foster inclusiveness and participation of previously marginalized actors in global governance. Figure 2 illustrates this continued marginalization of key stakeholders (in particular the UN major groups) by showing the number of partners from a specific sector in the total partnership sample (Bäckstrand 2012, pp. 252–253). So far, critics to the partnership approach arguing that it is simply a tool for powerful actors to consolidate power seem vindicated. However, as Fig. 3 indicates, since a majority of partnerships are led by international organizations and state agencies and not by business actors, the partnership approach cannot easily be subsumed under a “privatization of governance” framing.

Third, the analysis finds that most partnerships appear to lack the organizational capacity, resources, and transparency to implement their goals. A mere 15 % indicate a budget plan, 23 % have office space, 30 % seem to have staff members, and 5 % disclose a memorandum of understanding (Pattberg et al. 2012, pp. 257–258).

Fourth, multistakeholder partnerships are “not just neutral instruments for implementing internationally accepted sustainability norms, such as the Millennium Development Goals and Agenda 21, but rather sites of contestation over distinct technologies and practices” (Mert and Chan 2012, p. 40). On the contrary, some act as vehicles for controversial technologies—including nuclear energy, biotechnologies, biofuels, PVC, and vinyl—to gain UN-level recognition to name a few.

The limited success of multistakeholder partnerships in terms of problem-solving leaves room for improvements. In the next section, we discuss nine conditions for improved performance.

## Nine conditions for successful multistakeholder partnerships

As discussed above, the performance of multistakeholder partnerships can be improved. To this end, we have identified nine conditions for success frequently encountered in the literature on multistakeholder partnerships. First, the relevance of actors and their specific resources, identities, and histories; Second, the relevance of process management: and third, the relevance of the problem-structure and broader “situational context” (Visseren-Hamakers et al. 2007). Table 1 provides an overview of the features of the nine conditions for success and the key literature used throughout this survey, which supports the observations.

**Table 1:** Selection of key literature per condition for success

Conditions for success	Key literature
Actors	
1. Optimal partner mix	Beisheim (2012), Newell et al. (2012) and Gray (2007)
2. Effective leadership	Glasbergen (2010), Abbott and Snidal (2010) and Gray (2007)
Process	
3. Stringent goal-setting	Liese and Beisheim (2011), Abbott et al. (2000) and Keohane and Victor (2011)
4. Sustained funding	Martens (2007), and Reinicke et al. (2000)
5. Professional process management	Liese and Beisheim (2011), Szulecki et al. (2011), Aylward et al. (2003)
6. Regular monitoring, reporting, and evaluation to support organizational learning	Wigell (2008) and Bäckstrand (2012)
Context	
7. Active meta-governance	Biermann et al. (2009), Derkx and Glasbergen (2014) and Glasbergen et al. (2007)
8. Favorable political and social context	Stringer et al. (2014) and Beisheim and Liese (2014)
9. Fit to problem-structure	Miles et al. (2001), Abbott (2012) and Keohane and Victor (2011)

The following sections elaborate on each of the conditions and provide recommendations for decision-makers.

### Actors

#### Partners

Multistakeholder partnerships are essentially manifestations of networked governance which in turn is characterized by resource exchange. To find the appropriate mix of resources, knowledge and capabilities are thus by definition necessary to exploit synergies and an effective division of labor. For such networked governance to succeed, one needs the combined willingness, capability, and resources of partners, in particular, engagement from the most powerful and influential members (Beisheim 2012; Newell et al. 2012). Moreover, creating an optimal mix requires attention in the partner-search, and omitting powerful and important stakeholders can lead to suboptimal performance (Gray 2007, p. 36; Wigell 2008). A particularly salient issue was frequently raised as a topic during our consultations with CSOs, namely, the challenge of power-asymmetries between members. Large power-asymmetries in terms of sheer financial and human resources and information can be detrimental to trust among members from different sectors of society. To address partner challenges, it could be useful to map partners’ values and identities; to devise counter-arguments and common points of interest; and to identify where bargaining could be successful and not successful. Finally, transparency is key to building trust and mitigating power-asymmetries and should thus be promoted by engaging the members in open and transparent communication, decision-making, and evaluation.

To enable an optimal mix of partners, it is necessary to conduct a detailed and thorough assessment of needs (what partners would be needed to induce change?) and match it with the prevalent values and identities of potential partners.

### Leadership

Leadership by both individuals and organizations is by many considered a key ingredient, and during the course of the partnership’s life-time, different types of leadership are needed. The start of a partnership needs an entrepreneur or broker (Glasbergen 2010), “convener” (Gray 2007), or “orchestrator” (Abbott and Snidal 2010). They fill the functions of bringing people to the table, mitigating diverging opinions, and driving the difficult start-up process forward.

While good leadership is recognized as an important feature of successful partnerships, it remains difficult to operationalize. Most observers simply note that leadership is essential yet provide little information on the conditions for effective leadership and means to foster it. Nevertheless, it remains critical to identify and manage the different types of leadership needed for the partnership to succeed.

### Process

#### Goal-setting

Beisheim and Liese argue that the effectiveness of multistakeholder partnerships partly depends on the “precision of norms” (2011) or, in other words, on how ambitious and stringent the goals have been set. High levels of precision limit the room for interpretation, while lower degrees of precision allow for discretion and interpretation. In many cases, rules are so vague and broad that they impede compliance, monitoring, reporting, and evaluation, and consequently limit accountability and transparency. Precise rules and goals also have a stabilizing and reassuring effect on governments and firms to invest resources when trying to achieve the goals of the partnership (Keohane and Victor 2011). An emerging challenge is to foster coherence in the international norm system to avoid, what Biermann et al. have called “conflictive fragmentation” (2009). Goals should therefore be aligned with international norms. Finally, Visseren-Hamakers et al. also connect trust building and improved collaboration to the level of consensus regarding strategies and goals, which in turn increases the likelihood for success (2007, p. 163). Hence, goal-setting is not only about the end product but also the way in which goals were set in a collaborative and inclusive process.

For goal-setting to succeed, it is important to have a good process in place. This includes developing a common vision and goals from the very outset, working toward a common problem-definition, and aiming for the clear and measurable goals. Moreover, a mapping of the compatibility between a partnership’s goals and other related processes (e.g., SDGs, CSR, human rights) reduces the risk of conflictive fragmentation (Biermann et al. 2009).

#### Funding

As the governance of sustainable development is arguably moving away from multilateral treaties and implementation via state-based agencies and programs, authors have warned against more unstable streams of funding as financing is increasingly provided through voluntary and “ultimately unpredictable” goodwill from private financiers (Martens 2007, p. 5). However, there is little evidence that governments are more likely to sustain a constant stream of funding than, for example, private funders such as foundations, and it is plausible to think that funding shortages can be actively managed. Governments are by no means the only source of income since, while private initiatives and foundations are becoming wealthier and perhaps increasingly important for providing common goods, as is the case, for example, in global health governance. It nevertheless highlights the need for adequate funding. Sourcing funds has thus become an increasingly important task for managers. There is no template for what funding model that works best. Successful organizations have employed a number of approaches, for example, limiting funding coming from one source, relying on membership fees or voluntary funding from the members, and funnelling money generated from activities back to the organizations (Reinicke et al. 2000). In sum, securing funding is more of an issue for multistakeholder partnerships than for traditional implementation programs.

#### Management

A robust finding of partnership research is that effectiveness and good process management are related (Liese and Beisheim 2011). While it is hardly surprising that effective and efficient internal organization is conducive for the organizational goals to be met, in many cases, inadequate resources, time, and thinking are spent on managerial aspects. While the verdict is still out on what type of governance structure that optimizes effectiveness, some studies indicate that a small governing board of major donors, supported by a secretariat and room for input by a select group of affected stakeholders, is favorable for a lean and effective process management and decision-making (Liese and Beisheim 2011). Common strategic plans, clear division of roles and responsibilities, and multilevel forums to coordinate funding and resources have been identified as effective management structures (Aylward et al. 2003). Also smart management measures taken on a local level are found to facilitate success.

A frequent observation is that having full-time staff employed is conducive to effectiveness (Szulecki et al. 2011; Beisheim 2012). According to these studies, a high level of institutionalization with formal organization and bureaucracy is thus preferable to a loosely coupled network structure with, for example, a hosted secretariat within an already existing organization. However, there needs to be a balance between the level of institutionalization and the amount of red-tape. Existing institutions should be used as far as possible to avoid becoming a new institution or agency by limiting their bureaucracy’s work to only the essential coordination tasks (Reiniecke et al. 2000). Szulecki et al. also find that organizational characteristics such as a strong ‘corporate identity’ appear to be correlated with effectiveness (2011).

A good management structure includes staff focusing exclusively on partnership tasks, hiring staff with managerial experience to occupy key positions, ensuring effective communication between the process managers and key partnership members, as well as among the partnership members. Moreover, to avoid internal conflicts, we recommend creating dispute-settlement mechanisms.

### Monitoring, reporting, evaluation, and learning

Monitoring, reporting, and evaluation practices among different multistakeholder partnerships vary substantially. While some arrangements publicly disclose annual reports, third party evaluations, and meeting documents, others barely report on meeting agendas and participants. However, we argue that a robust and open monitoring, reporting, and evaluation system to record progress and processes will have a positive effect on the performance of multistakeholder partnerships for three main reasons. First, it enables organizational learning. Institutions have proven more effective when they are able to adapt quickly to new circumstances (Folke et al. 2005). Second, both public and private constituencies are increasingly demanding accountability and disclosure of spending and impacts of financial or in-kind contributions. Third and finally, monitoring, reporting, and evaluation are needed to enhance transparency, which in turn is instrumental for process legitimacy (Wigell 2008; Bäckstrand 2012; Gupta and Mason 2014).

Consequently, a transparent and regular monitoring and reporting program is conducive and even necessary to foster organizational learning and legitimation for the partnership’s raison d’être.

### Context

#### Meta-governance

A surge in alternative governance arrangements such as multistakeholder partnerships outside the traditional international institutions is an indicator of an emerging property of fragmentation in global governance which is characterized by uncoordinated and non-hierarchical institutional arrangements, often leading to functional overlap and competition among initiatives and norms (Biermann et al. 2009). Fragmentation could have negative effects on the governance architecture in the shape of inefficiencies and conflicting norms, goals, and policy processes. To mitigate the risk of “conflictive fragmentation”, multistakeholder partnerships should consider meta-governance, i.e., “the organisation of self-organisation” or “regulation of self-regulation” (Derkx and Glasbergen 2014). Research highlights two important aspects. First, goals of multistakeholder partnerships should be checked against a number of criteria to determine their conduciveness to, for example, the key principles of the UNFCCC, the Convention on Biological Diversity (CBD), the Hyogo Framework for Action, and other international policy goals. Second, if they are to be incorporated into the formal regime and given a “seal of approval” from the UN, then there should be a bureaucracy with the mandate and power to vet new initiatives against set criteria, in particular to avoid what has been described as “blue washing.”

To promote good meta-governance, goals should be checked against a number of minimum criteria for their conduciveness to the SDGs and other sustainable development-related goals (e.g., climate change 2° target). We also recommended to carefully map the broader governance architecture in which the partnership is situated and consequently liaison with other partnerships, organizations and institutions working with related problems.

#### Political and social context

Multistakeholder partnerships interact with numerous international, national, and local institutional frameworks with an impact on sustainable development, and thus add to a dense patchwork of existing institutions. Consequently, the political and social context will influence the chance to succeed. For example, national production and consumption patterns, views of ruling elites, or geographical position could be major factors determining the outcome of a partnership. The political and social context is important at, at least, two levels. First, the political and social context is relevant at the level of the actual governance architecture. Building on Visseren-Hamakers et al. we note that partnerships can have three functions vis-à-vis the wider governance architecture: if functions are filled that support multilateral regimes, they are complementary; if functions are filled that used to be carried out by governments, they erode public authority; and, if functions are fulfilled in a new manner, they reinvent politics (Visseren-Hamakers et al. 2012). Second, those partnerships with implementation at the local level are highly dependent on local conditions. This can be used to complement the benefits of the governance arrangement. For example, best practices from multistakeholder partnerships in developing countries show the importance of learning and building on local institutional and governance structures when delivering common goods. It has also been shown that institutional capacity building was needed, in particular, in countries with a violent past (Stringer et al. 2014).

Mapping the governance architecture and the social and political context in which a multistakeholder partnership is situated is central to understanding the opportunities and challenges to implementation. It increases the possibility for tailor-made solutions rather than a “one-size fits all” approach. In some cases, local capacity building to create the institutional conditions for implementation, taking into account local conditions, is a necessary strategy to pave the way for a successful arrangement (Beisheim and Liese 2014, 208).

#### Problem-structure

A final intervening variable that determines the likelihood of a successful partnership is the structure of the problem at hand. A range of researchers have argued that “malign problems” characterized by high levels of complexity, competing interests, and unclear solutions are less likely to be solved than “benign problems” where actors’ interests and preferences converge, and solutions are easier to identify (e.g., Miles et al. 2001). It is thus important to control for problem-structure when measuring the success of a partnership. In addition, when designing a partnership, it is therefore important to recognize that every problem has distinct features with specific administrative problems and political constituencies and thus requires different institutional setups (Abbott 2012; Keohane and Victor 2011). Problem-structure may, however, be malleable. Scientific discovery might reduce uncertainties in deciding what measures would be appropriate and thereby assist in building a business-case for addressing a policy issue.

Ultimately, it is important to investigate whether a multistakeholder partnership is the most appropriate solution to the problem at hand, or if there are other, more promising avenues that can be explored.

## Conclusions

In this perspective, we provided guidance on how to improve the performance of transnational multistakeholder partnerships for sustainable development by learning from past experience. It is a salient topic, as multistakeholder partnerships are increasingly utilized not only to implement global sustainable development goals such as the SDGs but also to feature prominently in adjacent issues areas such as climate change, biodiversity, and natural disasters.

Nine conditions have emerged from our analysis determining the success of a multistakeholder partnership. It suggests that in future design, implementation, or evaluation of partnerships, these aspects should be considered and taken into account throughout all stages of the partnership’s process. For instance, the problem-structure and social and political contexts will determine whether partnerships are the best means of implementation for the issue at hand. In the start-up phase of a partnership, entrepreneurial leadership and a proper goal-setting process is therefore needed. In addition, transparent procedures, adequate management skills, active monitoring and reporting, and sustained funding and feedback-loops for higher-level learning are essential for creating success.

The findings reported in this perspective also point to a number of shortcomings in existing research, and consequently indicate directions for future research. First, the temporal dynamics of transnational partnerships have not received sufficient attention. How is the universe of multistakeholder partnerships changing over time? Which arrangements serve only strategic purposes and which are further institutionalized into solid organizations? Who is driving changes over time? A second research lacuna is the question of how the nine conditions for success interact with each other. Are there conceivable trade-offs between the conditions? Are certain combinations more important than others? And finally, empirical research should scrutinize whether some conditions are more or less important for a specific type of partnership.

On a final note, while the logic behind transnational multistakeholder partnership is attractive for addressing complex sustainable development problems, they have yet to reach their full potential. Moreover, the failure to significantly enhance participation and inclusiveness in global governance through multistakeholder partnerships also provide critics with evidence of their “dark side.” However, research could help in identifying areas for improvement and potential pitfalls and provide an evidence-based review for a way forward. Over the coming year(s), there will be important steps to be made toward implementing the SDGs, reversing biodiversity loss and deforestation, and mitigate greenhouse gases. And there are already calls and proposals for changing the meta-governance of partnerships to improve their effectiveness. For instance, Chan and Pauw (2014)—supported by a group of scholar and think-thanks—have suggested a Global Framework for Climate Action (GFCA) to address many of the shortcomings observed in the sustainable development arena. A GFCA would support the mobilization of alternative governance arrangements such as subnational and city initiatives on climate change, by providing brokerage, visibility, and legitimacy. In return, arrangements such as multistakeholder partnership would be required to report on their progress and be subjected to more monitoring than presently the case. Chan and Pauw’s (2014) suggestion is a laudable attempt to improve the overall global governance of sustainable development issues and could be seen as an indication of how the international arena is trying to deal with suboptimal performance of alternative governance arrangements. However, these calls for more overall synergies can only be realized if the building blocks, i.e., the individual governance arrangements, are designed and implemented in ways that enable their success. We hope that our discussion of nine conditions for success can provide such guidance for improving the performance of multistakeholder partnerships.

## Footnotes

1 http://sustainabledevelopment.un.org/index.php?menu=1500.

## References

[CR300] Abbott, K.W. 2012. Engaging the public and the private in global sustainability governance. *International Affairs* 88: 543–564.

[CR1] Abbott KW, Snidal D (2010). International regulation without international government: Improving international organization performance through orchestration. The Review of International Organizations.

[CR500] Abbott, K.W., R.O. Keohane, A. Moravcsik, A.-M. Slaughter, and D. Snidal. 2000. The concept of legalization. *International Organization* 54: 401–419.

[CR2] Andonova LB (2010). Public–private partnerships for the earth: Politics and patterns of hybrid authority in the multilateral system. Global Environmental Politics.

[CR3] Andonova LB, Levy MA (2003). Franchising global governance: Making sense of the Johannesburg type II partnerships. Yearbook of International Co-operation on Environment and Development.

[CR4] Aylward RB, Acharya A, England S, Agocs M, Linkins J (2003). Global health goals: Lessons from the worldwide effort to eradicate poliomyelitis. The Lancet.

[CR5] Bäckstrand K, Pattberg P, Biermann F, Chan S, Mert A (2012). Are partnerships for sustainable development democratic and legitimate?. Public–private partnerships for sustainable development: Emergence, influence and legitimacy.

[CR6] Beisheim, M. 2012. *Partnerships for sustainable development: why and how Rio* + *20 must improve the framework for multi*-*stakeholder partnerships*. RP 3. Stiftung Wissenschaft und Politik, German Institute for International and Security Affairs.

[CR7] Beisheim M, Liese A (2014). Transnational partnerships: Effectively providing for sustainable development?.

[CR8] Biermann F, Pattberg P, Van Asselt H, Zelli F (2009). The fragmentation of global governance architectures: A framework for analysis. Global Environmental Politics.

[CR9] Biermann F, Abbott KW, Andresen S, Bäckstrand K, Bernstein S, Betsill MM, Bulkeley H, Cashore B (2012). Navigating the Anthropocene: Improving earth system governance. Science.

[CR10] Bitzer V, Francken M, Glasbergen P (2008). Intersectoral partnerships for a sustainable coffee chain: Really addressing sustainability or just picking (coffee) cherries?. Global Environmental Change.

[CR11] Börzel TA, Risse T (2005). Public–private partnerships: Effective and legitimate tools of transnational governance?.

[CR12] Brinkerhoff DW, Brinkerhoff JM (2011). Public–private partnerships: Perspectives on purposes, publicness, and good governance. Public Administration and Development.

[CR13] Bruno, K., and J. Karliner. 2000. Tangled up in blue: Corporate partnerships at the United Nations. San Francisco: CorpWatch.

[CR14] Chan, S., and P. Pauw. 2014. *Proposal for a global framework for climate action to engage non*-*state and subnational stakeholders in the future climate regime*. Briefing paper 15/2014. German Development Institute.

[CR15] Cole DH (2015). Advantages of a polycentric approach to climate change policy. Nature Climate Change.

[CR16] Derkx B, Glasbergen P (2014). Elaborating global private meta-governance: An inventory in the realm of voluntary sustainability standards. Global Environmental Change.

[CR17] Folke C, Hahn T, Olsson P, Norberg J (2005). Adaptive governance of social–ecological systems. Annual Review of Environment and Resources.

[CR18] Glasbergen P (2010). Global action networks: Agents for collective action. Adaptive capacity to global change in Latin. Global Environmental Change.

[CR19] Glasbergen P, Biermann F, Mol APJ (2007). Partnerships, governance and sustainable development: Reflections on theory and practice.

[CR20] Gray, B. 2007. The process of partnership construction: Anticipating obstacles and enhancing the likelihood of successful partnerships for sustainable development. In *Partnerships, Governance and Sustainable Development. Reflections on Theory and Practice*, ed. P. Glasbergen, F. Biermann, and A.P.J. Mol, 27–41. Cheltenham: Edward Elgar.

[CR21] Gupta A, Mason M, Betsill MM, Hochstetler K, Stevis D (2014). Transparency and international environmental politics. Advances in international environmental politics.

[CR22] Hajer M, Nilsson M, Raworth K, Bakker P, Berkhout F, de Boer Y, Rockström J, Ludwig K (2015). Beyond cockpit-ism: Four insights to enhance the transformative potential of the sustainable development goals. Sustainability.

[CR23] Hale TN, Mauzerall DL (2004). Thinking globally and acting locally: Can the Johannesburg partnerships coordinate action on sustainable development?. The Journal of Environment & Development.

[CR24] Keohane RO, Victor DG (2011). The regime complex for climate change. Perspectives on Politics.

[CR302] Liese, A., and M. Beisheim. 2011. Transnational public-private partnerships and the provision of collective goods in developing countries. In *Governance without a state? policies and politics in areas of limited statehood*, ed. T. Risse, 115–143.

[CR25] Martens J (2007). Multistakeholder partnerships: Future models of multilateralism.

[CR26] Mert A, Chan S, Pattberg P, Biermann F, Chan S, Mert A (2012). The politics of partnerships for sustainable development. Public–private partnerships for sustainable development. Emergence, influence and legitimacy.

[CR303] Miles, E.L., A. Underdal, S. Andresen, J. Wettestad, J.B. Skjaerseth, and E.M Carlin. 2001. *Environmental regime effectiveness: Confronting theory with evidence*. Cambridge: The MIT Press.

[CR27] Newell P, Pattberg P, Schroeder H (2012). Multiactor governance and the environment. Annual Review of Environment and Resources.

[CR28] Pattberg P (2005). The Forest Stewardship Council: Risk and potential of private forest governance. Journal of Environment & Development.

[CR29] Pattberg P, Glasbergen P, Biermann F, Mol A (2007). Partnerships for sustainability? An analysis of transnational environmental regimes. Partnerships, governance and sustainable development. reflections on theory and practice.

[CR30] Pattberg P (2010). Public–private partnerships in global climate governance. Wiley Interdisciplinary Review: Climate Change.

[CR310] Pattberg, P., and A. Mert. 2013. The future we get might not be the future we want: Analyzing the Rio+20 outcomes. *Global Policy* 4: 305–310.

[CR304] Pattberg, P., F. Biermann, S. Chan, and A. Mert. 2012. *Public-private partnerships for sustainable development: Emergence, influence and legitimacy*. Cheltenham: Edward Elgar.

[CR31] Reinicke WH, Deng F, Witte JM (2000). Critical choices: The United Nations, networks, and the future of global governance.

[CR32] Rockström J, Steffen W, Noone K, Persson A, Chapin FS, Lambin E, Lenton TM, Scheffer M (2009). Planetary boundaries: Exploring the safe operating space for humanity. Ecology and society.

[CR33] Schäferhoff M, Campe S, Kaan C (2009). Transnational public–private partnerships in international relations: Making sense of concepts, research frameworks, and results. International Studies Review.

[CR34] Stringer LC, Dougill AJ, Dyer JC, Vincent K, Fritzsche F, Leventon J, Falcão MP, Manyakaidze P (2014). Advancing climate compatible development: Lessons from southern Africa. Regional Environmental Change.

[CR35] Szulecki K, Pattberg P, Biermann F (2011). Explaining variation in the effectiveness of transnational energy partnerships. Governance.

[CR36] UNCED. 1992. *AGENDA 21*. United Nations Conference on Environment and Development. Rio de Janerio: United Nations Conference on Environment and Development. 10.1038/nbt0492-402 1368483

[CR37] UNFCCC. 2014. Portal on cooperative initiatives.

[CR38] UNFCCC. 2015. The Non-state Actor Zone for Climate Action (NAZCA).

[CR39] Visseren-Hamakers IJ, Arts B, Glasbergen P (2007). Partnership as governance mechanism in development cooperation: Intersectoral north–south partnerships for marine biodiversity.

[CR40] Visseren-Hamakers IJ, Leroy P, Glasbergen P (2012). Conservation partnerships and biodiversity governance: Fulfilling governance functions through interaction. Sustainable Development.

[CR41] Vollmer D (2009). Enhancing the effectiveness of sustainability partnerships: Summary of a workshop.

[CR42] Widerberg O, Pattberg P (2015). International Cooperative Initiatives in global climate governance: Raising the ambition level or delegitimizing the UNFCCC?. Global Policy.

[CR43] Wigell M (2008). Multi-stakeholder cooperation in global governance.

[CR44] Zammit A (2003). Development at risk: Rethinking UN-business partnerships.

[CR45] Zelli F (2011). The fragmentation of the global climate governance architecture. Wiley Interdisciplinary Reviews: Climate Change.

